# Antifungal Activity of *Trichoderma asperellum* ZZY Fermentation Products Against Postharvest Cherry Tomato Pathogens and Their Preservation Effect

**DOI:** 10.3390/foods15203682

**Published:** 2026-10-16

**Authors:** Qi Zhu, Haoyu Wang, Tengda Li, Sijie Zhu, Xianwei Zhang, Chong Sun, Huiqing Sun, Zhenyuan Zhu

**Affiliations:** 1 State Key Laboratory of Food Nutrition and Safety, Tianjin University of Science and Technology, Tianjin 300457, China; zhuqi9622@163.com (Q.Z.); wwwhyyy1@163.com (H.W.); 13194653036@163.com (T.L.); 18798006458@163.com (S.Z.); 15933701492@163.com (X.Z.); 15613402005@163.com (C.S.); sunhuiqing@tust.edu.cn (H.S.); 2 Key Laboratory of Food Nutrition and Safety, Ministry of Education, Tianjin University of Science and Technology, Tianjin 300457, China; 3 College of Food Science and Engineering, Tianjin University of Science and Technology, Tianjin 300457, China

**Keywords:** *Trichoderma asperellum* ZZY, antifungal activity, TAFLP, cherry tomato, preservation effect

## Abstract

Postharvest fungal diseases cause substantial losses in fresh produce, highlighting the need for effective and environmentally compatible alternatives to conventional fungicides. This study investigated the antifungal activity of *Trichoderma asperellum* ZZY against *Botrytis cinerea*, *Alternaria alternata*, and *Fusarium oxysporum*, as well as its potential for cherry tomato preservation. Confrontation, volatile, and non-volatile assays demonstrated that ZZY inhibits these pathogens through multiple antagonistic mechanisms. The fermentation filtrate exhibited strong antifungal activity, with the highest activity retained in the aqueous fraction after solvent partitioning. Biochemical characterization of the proteinaceous component TAFLP and enzymatic assays indicated chitinolytic activity, while HS-SPME-GC-MS analysis identified diverse volatile compounds that may contribute to antifungal effects. Furthermore, treatment with the fermentation filtrate reduced decay and disease development in cherry tomatoes during storage while helping maintain fruit quality. These findings link the antagonistic activity of *Trichoderma asperellum* ZZY with its fermentation-derived antifungal components and postharvest preservation effects, supporting further investigation of ZZY-derived products as potential biocontrol agents for postharvest disease management.

## 1. Introduction

Fruits and vegetables are essential components of the human diet and are rich in vitamins, minerals, antioxidants, and other nutrients that are closely associated with human health [1,2]. However, during postharvest handling, transportation, and storage, diseases and mechanical damage can substantially impair the quality of fresh produce and result in considerable economic losses [3,4]. Fungal pathogens, including *Botrytis cinerea*, *Alternaria* spp., and *Fusarium* spp., are among the major causes of postharvest decay and can accelerate tissue deterioration and nutrient loss in fruits and vegetables such as tomatoes and grapes [5,6]. In addition, some postharvest fungal pathogens can produce mycotoxins, thereby posing additional risks to food safety [7,8]. Currently, synthetic fungicides remain an important approach for controlling postharvest fungal diseases because of their rapid action and relatively high efficacy. However, the long-term and repeated use of chemical fungicides may lead to environmental contamination, pesticide residues, the development of fungicide resistance in pathogens, and potential health risks associated with prolonged exposure [9,10,11]. Therefore, increasing attention has been devoted to the development of safe and effective biological control strategies as alternatives or complementary approaches to conventional chemical fungicides.

Currently, a variety of microorganisms, including *Bacillus*, *Pseudomonas*, *yeasts*, and *Trichoderma*, have been investigated and applied for the biological control of plant pathogens [12,13,14]. Among these microorganisms, fungi of the genus *Trichoderma* have been extensively studied as biocontrol agents because of their diverse and complementary antagonistic mechanisms. *Trichoderma* can suppress the growth of plant pathogenic fungi through multiple mechanisms, including competition for nutrients and space, direct mycoparasitism, production of cell wall-degrading enzymes, and secretion of volatile and non-volatile antimicrobial metabolites [15,16,17]. During direct interactions with pathogens, *Trichoderma* hyphae can recognize, attach to, and coil around the hyphae of pathogenic fungi, followed by penetration and localized constriction and cellular damage of the pathogen hyphae [18]. In addition, extracellular hydrolytic enzymes, such as chitinases, β-glucanases, and proteases, can degrade structural components of fungal cell walls, thereby compromising cell wall integrity and inhibiting fungal growth [19,20,21]. Meanwhile, *Trichoderma* can produce volatile organic compounds (VOCs) that diffuse through the surrounding environment and inhibit pathogen growth in the absence of direct physical contact [22,23]. These diverse and complementary mechanisms make *Trichoderma* a promising candidate for the biological control of postharvest fungal diseases.

Previous studies have provided substantial evidence supporting the potential of *Trichoderma* for the control of postharvest and soil-borne fungal pathogens. For example, *Trichoderma asperelloides* SKRU-01 and its fermentation broth were reported to inhibit the growth of *Penicillium alaccae*, with the pathogen hyphae being penetrated and surrounded by *Trichoderma* hyphae, followed by cytoplasmic disruption. Treatment with the fermentation broth also delayed the decay of snake fruit [24]. The aspartic protease P6281 from *Trichoderma harzianum* exhibits broad-spectrum antifungal activity and effectively controls postharvest gray mold, highlighting its potential as a biocontrol agent for fruit and vegetable preservation [25]. Other studies have demonstrated that *Trichoderma* strains can suppress *Fusarium* spp. through volatile compounds and effectively inhibit the growth of pathogens belonging to genera such as *Botrytis*, *Rhizoctonia* and *Sclerotinia* [26]. These findings highlight the broad potential of *Trichoderma* as a biological control agent. However, the antifungal efficacy of *Trichoderma* is highly dependent on factors such as the fungal strain, target pathogen, cultivation conditions, and experimental evaluation system. More importantly, existing studies have often focused separately on the direct antagonistic activity of living *Trichoderma* or on individual antimicrobial metabolites, whereas the respective contributions and potential interactions of direct contact-dependent antagonism, volatile compounds, and non-volatile extracellular factors produced by the same *Trichoderma* strain remain insufficiently characterized. In particular, the relationships among the antagonistic activity of living *Trichoderma*, the antifungal activity of its fermentation filtrate, the characteristics of proteinaceous active components in the fermentation filtrate, and the extent to which these activities translate into practical postharvest preservation effects remain to be fully elucidated.

Therefore, further investigation is needed to clarify the antagonistic mechanisms of *Trichoderma asperellum* and its relationships with fermentation-derived antifungal components, as well as its potential application in postharvest disease management. In this study, *Trichoderma asperellum* ZZY was evaluated for its antagonistic activity against three important postharvest fungal pathogens, *Botrytis cinerea*, *Alternaria alternata*, and *Fusarium oxysporum*. The specific objectives were to: (i) characterize the contact-dependent, volatile, and non-volatile antagonistic activities of ZZY against these pathogens; (ii) evaluate the antifungal activity of the ZZY fermentation filtrate and characterize its active components through solvent fractionation and biochemical analyses, with particular attention to its proteinaceous component, designated as *Trichoderma asperellum* fermentation liquor protein (TAFLP); and (iii) assess the potential of the fermentation filtrate to reduce postharvest decay and maintain the quality of cherry tomatoes during storage. By integrating the evaluation of antagonistic mechanisms, fermentation-derived antifungal components, and postharvest preservation effects, this study provides insights into the biocontrol potential of *Trichoderma asperellum* ZZY and its fermentation products for postharvest disease management.

## 2. Materials and Methods

### 2.1. Strain Activation and Cultivation

The *Trichoderma asperellum* ZZY strain was previously isolated from soil by our laboratory and identified as *Trichoderma asperellum* based on colony and morphological characteristics combined with homology analysis of the ITS rDNA sequence. The strain is currently deposited at the China General Microbiological Culture Collection Center (CGMCC) under the accession number CGMCC 12071. The pathogenic fungi *Botrytis cinerea*, *Alternaria alternata* and *Fusarium oxysporum* were provided by the laboratory of Professor Honglian Guo at Tianjin University of Science and Technology. All three pathogens had been previously identified and maintained as laboratory strains and were used as known plant pathogenic fungi in the subsequent antagonistic and postharvest preservation experiments. All four fungal strains used in this study were maintained on PDA slants at 4 °C. For activation and subculturing, fungal plugs with a diameter of 6 mm were aseptically excised from the PDA slants using a sterile cork borer and transferred onto sterile PDA plates. The plates were incubated at 26 ± 0.5 °C. After 3–5 days of incubation, well-developed and contamination-free mycelial plugs were selected and transferred onto fresh sterile PDA plates. After three consecutive subcultures, fungal cultures showing good growth and high purity were obtained and used for subsequent experiments [27].

### 2.2. Evaluation of the Antagonistic Activity of Trichoderma asperellum ZZY Against Postharvest Pathogenic Fungi

#### 2.2.1. In Vitro Confrontation Assay

The confrontation assay was performed according to the method reported by Geiger et al. [28]. Mycelial plugs with a diameter of 6 mm were aseptically excised using a sterile cork borer from 5-day-old potato dextrose agar (PDA) cultures of *Trichoderma asperellum* ZZY and the three pathogenic fungi, *Botrytis cinerea*, *Alternaria alternata*, and *Fusarium oxysporum*. For the confrontation assay, a mycelial plug of ZZY and a mycelial plug of each pathogen were simultaneously inoculated onto the same 90-mm PDA plate. The two plugs were placed along the diameter of the plate, with a center-to-center distance of approximately 30 mm. The plates were incubated at 26 ± 0.5 °C for 5 days, after which the radial growth distance of each pathogen toward the ZZY colony was measured and recorded. PDA plates inoculated with only the corresponding pathogen under the same incubation conditions served as controls. Three independent replicates were included for each treatment. The antagonistic activity of ZZY against *Botrytis cinerea*, *Alternaria alternata*, and *Fusarium oxysporum* was evaluated by comparing the radial growth of the pathogens in the confrontation assay with that in the corresponding pathogen-only controls. The inhibition rate was calculated using Formula (1).

(1) $$\mathrm{IR}=\frac{A\;-\;B}{A}\times 100\%$$

where *IR* is the inhibition rate (%), *A* is the radial growth of the pathogen in the control group (cm), and *B* is the radial growth of the pathogen toward the ZZY colony in the confrontation group (cm).

#### 2.2.2. Cellophane Overlay Assay

A sterile cellophane membrane (90 mm in diameter and approximately 25 μm in thickness, Solarbio, Beijing, China) was placed flat over the entire surface of a PDA plate to prevent direct contact between the fungal mycelium and the agar medium. A mycelial plug of *Trichoderma asperellum* ZZY (6 mm in diameter) was placed at the center of the cellophane membrane and incubated at 26 ± 0.5 °C for 3 days. During incubation, ZZY was allowed to grow on the cellophane membrane, while the cellophane membrane served as a physical barrier between the developing ZZY colony and the PDA medium. After 3 days of incubation, the entire cellophane membrane together with the ZZY colony growing on it was carefully removed from the PDA plate. Subsequently, a 6-mm-diameter mycelial plug of the corresponding pathogenic fungus was placed at the center of the same PDA plate and incubated at 26 ± 0.5 °C for an additional 3 days [29]. The radial growth of the pathogen colony was then measured. PDA plates inoculated with the corresponding pathogen without prior incubation of ZZY on the cellophane membrane were used as the control group. The inhibition rate was calculated according to Formula (1).

#### 2.2.3. Sandwich Plate Culture Assay

The mycelial block (φ = 6 mm) of ZZY was inoculated onto PDA plates and incubated until the colony reached a diameter of approximately 5 cm. The fungal block was then removed, and a sterile piece of cellophane was placed flat on the surface of the plate. Another PDA plate of the same size, containing a mycelial block (φ = 6 mm) of the pathogenic fungus, was then placed face to face with the prepared plate [30]. After 3 days of incubation at 26 ± 0.5 °C, the colony diameter was measured. Culture plates inoculated solely with the pathogenic fungus served as the control. The inhibition rate was calculated using Formula (1).

#### 2.2.4. Microscopic Observation of Antagonistic Interaction

Under sterile conditions, 1 mL of potato dextrose broth (PDB) medium was evenly spread onto a microscope slide to form a thin film. After cooling and solidification, mycelial blocks of ZZY and the pathogenic fungus were inoculated at opposite ends of the slide, approximately 3 cm apart. The slide was incubated in a sterile, constant-temperature incubator at 26 ± 0.5 °C for 3 days. When the two clones grew to the point of contact, the slide was removed and observed under a light microscope [31]. Images were captured to record the interaction.

### 2.3. Preparation of ZZY Fermentation Filtrate and Evaluation of Its Antifungal Activity and Stability

#### 2.3.1. Preparation of ZZY Fermentation Filtrate

Mycelial blocks were obtained from ZZY PDA plates using a sterile cork borer (Model 78633022, CHINOK, Jilin, China) and transferred into 100 mL of sterile PDB. They were incubated on a rotary shaker at 26 ± 0.5 °C and 150 rpm for 5 days. After incubation, the fermentation broth was centrifuged and filtered to separate the mycelia from the supernatant. The supernatant was then filtered through a sterile membrane filter to obtain a sterile fermentation filtrate, which was stored at 4 °C for later use [32].

#### 2.3.2. Antifungal Activity of the ZZY Fermentation Filtrate

A mycelial plug of *Trichoderma asperellum* ZZY (φ = 6 mm) was inoculated into sterile PDB and incubated at 26 ± 0.5 °C with shaking at 150 rpm. Fermentation broth samples were collected after 0, 1, 3, 5, and 7 days of cultivation. The fermentation broth was initially filtered to remove the mycelial biomass, followed by centrifugation at 4000 rpm for 15 min. The resulting supernatant was further sterilized by filtration through a sterile membrane filter to obtain the ZZY fermentation filtrate. After the PDA medium had cooled to room temperature but had not yet solidified, 20 mL of the fermentation filtrate was thoroughly mixed with 100 mL of PDA medium and poured into sterile Petri dishes. After the medium had completely solidified, a mycelial plug of the corresponding pathogenic fungus was inoculated at the center of each plate. PDA plates inoculated with the corresponding pathogen without the addition of fermentation filtrate were used as controls [33]. The plates were incubated at 26 ± 0.5 °C for 5 days, after which the colony diameter of each pathogen was measured. Three biological replicates were included for each treatment. The inhibition rate of the ZZY fermentation filtrate against pathogen mycelial growth was calculated according to Equation (1).

#### 2.3.3. Effect of Temperature Treatment on the Antifungal Activity of the ZZY Fermentation Filtrate

Aliquots of 25 mL of the sterile ZZY fermentation filtrate were transferred into sterile 50 mL conical flasks and subjected to heat treatment at 20, 40, 60, 80, and 100 °C in a water bath or at 121 °C in an autoclave (DSX-280KB24, Shanghai, China) for 30 min. After treatment, the fermentation filtrates were allowed to cool naturally to room temperature [34]. The antifungal activities of the treated fermentation filtrates against *Botrytis cinerea*, *Alternaria alternata* and *Fusarium oxysporum* were subsequently evaluated according to the procedure described in Section 3.2.

#### 2.3.4. Effect of Storage Time on the Antifungal Activity of the ZZY Fermentation Filtrate

Aliquots of sterile ZZY fermentation filtrate were stored at 4 °C and 26 °C for 1, 3, 7, 10, 20, and 30 days, respectively. After each designated storage period, the antifungal activity of the fermentation filtrate against *Botrytis cinerea* was determined according to the procedure described in Section 3.2 [35].

#### 2.3.5. Effect of UV Irradiation on the Antifungal Activity of the ZZY Fermentation Filtrate

In a laminar flow cabinet, 20 mL of sterile ZZY fermentation filtrate was transferred into sterile glass Petri dishes (90 mm in diameter). The dishes were placed 30 cm below a UV lamp (254 nm, 200 W) and irradiated for 2, 4, 6, and 8 h [36]. After each irradiation period, the antifungal activity of the treated fermentation filtrates against *Botrytis cinerea*, *Alternaria alternata*, and *Fusarium oxysporum* was evaluated according to the procedure described in Section 3.2.

#### 2.3.6. Effect of Oxidizing and Reducing Agents on the Antifungal Activity of the ZZY Fermentation Filtrate

Hydrogen peroxide (H_2_O_2_, Tianjin Jiangtian Chemical Technology Co., Ltd., Tianjin, China) was prepared in sterile water at final concentrations of 0.1%, 0.3%, 0.5%, and 1.0%, while sodium sulfite (Na_2_SO_3_, Tianjin Jiangtian Chemical Technology Co., Ltd., Tianjin, China) was prepared in sterile water at concentrations of 0.01, 0.03, 0.05, and 0.10 mol/L. Each H_2_O_2_ or Na_2_SO_3_ solution was mixed thoroughly with the sterile ZZY fermentation filtrate at a volume ratio of 1:9 [37]. The antifungal activities of the resulting fermentation filtrate treatments against *Botrytis cinerea*, *Alternaria alternata* and *Fusarium oxysporum* were subsequently evaluated according to the procedure described in Section 3.2.

#### 2.3.7. Effect of Metal Ions on the Antifungal Activity of the ZZY Fermentation Filtrate

Standard solutions of Mg^2+^, K^+^, Na^+^, Cu^2+^, and Fe^2+^ (Tianjin Jiangtian Chemical Technology Co., Ltd., Tianjin, China) at a concentration of 0.1 mg/mL were prepared and separately mixed with the sterile ZZY fermentation filtrate at a volume ratio of 1:9. After thorough mixing, the effects of the different metal ion treatments on the antifungal activity of the ZZY fermentation filtrate against *Botrytis cinerea*, *Alternaria alternata*, and *Fusarium oxysporum* were evaluated according to the procedure described in Section 3.2 [38].

### 2.4. Extraction, Characterization, and Enzymatic Activity Analysis of Antimicrobial Components from ZZY Fermentation Filtrate

#### 2.4.1. Stepwise Extraction of ZZY Fermentation Filtrate

A 50 mL aliquot of the ZZY fermentation filtrate was transferred into a 200 mL conical flask and extracted with 50 mL of petroleum ether (Tianjin Jiangtian Chemical Technology Co., Ltd., Tianjin, China) to obtain the lipophilic fraction. The flask was sealed and shaken at 150 rpm at room temperature for 12 h. The mixture was then transferred to a 100 mL separatory funnel and allowed to stand for several hours until complete phase separation occurred. The aqueous phase was collected and extracted three times with petroleum ether, and the petroleum ether phases were combined. The remaining aqueous phase was subsequently extracted three times with ethyl acetate (Tianjin Jiangtian Chemical Technology Co., Ltd., Tianjin, China), followed by three successive extractions with n-butanol (Tianjin Jiangtian Chemical Technology Co., Ltd., Tianjin, China). The petroleum ether, ethyl acetate, and n-butanol fractions were collected separately and concentrated under reduced pressure using a rotary evaporator. The residual aqueous phase was retained as the aqueous fraction. All fractions were stored for subsequent antifungal activity analysis [39].

#### 2.4.2. Analysis of Volatile Compounds in the Fermentation Broth

The ZZY fermentation broth was filtered through sterile membrane filter (0.22 μm), and the filtrate was transferred into a 15-mL solid-phase microextraction (SPME) vial. The vial was pre-equilibrated and heated in water bath at 60 °C for 20 min. Subsequently, an SPME fiber coated with 65 μm polydimethylsiloxane/divinylbenzene (PDMS/DVB) was inserted into the vial for headspace solid-phase microextraction. After extraction for 4 min, the SPME fiber was immediately inserted into the injection port of a gas chromatography-mass spectrometry (GC-MS, ISQ 7000, Thermo Fisher Scientific, Waltham, MA, USA) instrument and thermally desorbed at 250 °C for 5 min. The volatile compounds in the ZZY fermentation broth were subsequently analyzed [40].

#### 2.4.3. Fourier Transform Infrared (FTIR) Spectroscopy Analysis

Exactly 1.00 mg of TAFLP and 150.00 mg of KBr were accurately weighed and thoroughly ground together in a mortar until a fine and homogeneous powder was obtained. The mixture was then compressed into a pellet using a hydraulic pellet press (HY-12, Tianjin Tianguang New Optical Instrument Technology Co., Ltd., Tianjin, China). FTIR spectra were recorded using an FTIR spectrometer (TENSOR 27, Bruker, Karlsruhe, Germany) over the range of 400–4000 cm^−1^ [41].

#### 2.4.4. MALDI-TOF Mass Spectrometry Analysis

The experimental procedure was adapted from the method described by Dave et al. [42]. TAFLP was dissolved in ultrapure water and thoroughly mixed with a saturated solution of α-cyano-4-hydroxycinnamic acid (HCCA) matrix. The resulting mixture was air-dried at room temperature and subsequently analyzed by MALDI-TOF MS (Bruker, Karlsruhe, Germany) to determine the molecular mass of TAFLP.

#### 2.4.5. Circular Dichroism (CD) Spectroscopy Analysis

The TAFLP solution (0.1 mg/mL) was analyzed using a MOS-450 circular dichroism spectropolarimeter (Bio-Logic, Grenoble, France). CD spectra were recorded in a 0.1 cm pathlength quartz cuvette over the wavelength range of 190–260 nm, with a data interval of 1 nm and an acquisition time of 1 s per point. The mean residue ellipticity ([θ]) was expressed in deg·cm^2^·dmol^−1^ [43]. The secondary structure composition of TAFLP was estimated using the DichroWeb online analysis server (https://bio.tools/dichroweb), URL (accessed on 12 October 2023).

#### 2.4.6. Detection of Antimicrobial Protein Enzymatic Activity

The enzymatic activities of TAFLP were evaluated using an agar well diffusion assay [44]. PDA plates supplemented with 1% colloidal chitin (Tianjin Solomon Biotechnology Co., Ltd., Tianjin, China), 0.1% laminarin(Tianjin Jiangtian Chemical Technology Co., Ltd., Tianjin, China), 1% CMC-Na (Shanghai Yuanye Bio-Technology Co., Ltd., Shanghai, China), and 1% skim milk(ianjin Taijin Technology Co., Ltd., Tianjin, China) were prepared, and wells with a diameter of 9 mm were punched in the agar. Subsequently, 100 μL of the TAFLP solution was added to each well, and the plates were incubated at 27 °C in a constant-temperature incubator. After incubation, the plates containing laminarin and CMC-Na were stained with 0.1% Congo red solution for 5 min and then destained by removing the staining solution. The presence of clear hydrolysis zones was observed to assess the β-1,3-glucanase and cellulase activities of TAFLP. Protease activity was evaluated directly by observing the formation of clear hydrolysis zones on the skim milk plates without staining. For the detection of chitinase activity, the colloidal chitin plates were stained with 2% iodine solution, and the formation of clear hydrolysis zones was recorded.

### 2.5. Evaluation of the Postharvest Preservation Efficacy of Trichoderma asperellum ZZY Fermentation Broth on Cherry Tomatoes

#### 2.5.1. Fruit Firmness

Cherry tomatoes (cultivar ‘Millennium Fruit’) were purchased from Shouguang, Shandong, China. A total of 5 kg of fruits with similar maturity and size (individual fruit weight, 14 ± 1 g) and without visible mechanical damage or disease symptoms were selected for the subsequent experiments. The fruits were randomly and evenly divided into four groups: the control group (CK), fermentation broth treatment group (FJY), artificially wounded and inoculated group (SS), and artificially wounded and inoculated plus fermentation broth treatment group (SS + FJY). During the treatment period, six fruits were randomly selected from each group every 2 days for firmness measurements. For each fruit, two relatively opposite points on the equatorial region were selected as measurement sites. During measurement, the probe of a GY-4 fruit firmness tester was positioned vertically against the fruit surface and pressed into the fruit tissue at a constant speed. The firmness value was recorded after the reading stabilized [45]. After each measurement, the probe was returned to its initial position before the next measurement point was tested.

#### 2.5.2. Weight Loss Rate

The decay status of the cherry tomatoes in each treatment group was observed and recorded every 2 days, and the total weight of the fruits in each group was measured simultaneously [46]. Based on the changes in fruit weight during storage, the weight loss rate was calculated using the following equation.

$$\mathrm{Weight}\;\mathrm{loss}\;\mathrm{rate}\;\left(\%\right)=\frac{\;M_{0}\;-\;M}{M_{0}}\times 100\%$$

where *M*_0_ is the initial fruit weight before storage (g), and *M* is the fruit weight measured at each designated storage time (g).

#### 2.5.3. Color Changes

The surface color of the cherry tomatoes was measured every 2 days using a portable colorimeter (WR-10, Suzhou, China). The color parameters displayed by the instrument were recorded, including L* (lightness) and a* (redness/greenness) [47].

#### 2.5.4. Titratable Acidity (TA)

Titratable acidity was determined by the sodium hydroxide (NaOH, Tianjin Jiangtian Chemical Technology Co., Ltd., Tianjin, China) titration method according to a previously reported procedure [48]. Briefly, 0.25 g of cherry tomato samples, which had been frozen in liquid nitrogen and thoroughly pulverized, was accurately weighed and transferred to a 50 mL volumetric flask. Distilled water was added to the calibration mark, and the mixture was thoroughly shaken and allowed to stand for 30 min before filtration. Subsequently, 20 mL of the filtrate was transferred into a conical flask, and two drops of 1% phenolphthalein indicator were added. The filtrate was titrated with standardized 0.01 mol/L NaOH solution. The endpoint was reached when a pale pink color first appeared and persisted for 30 s without fading. The volume of NaOH solution consumed was recorded. Titratable acidity was expressed as malic acid content using a malic acid conversion factor of 0.067.

#### 2.5.5. Malondialdehyde Content

Malondialdehyde (MDA) content was determined using a commercial MDA assay kit (Solarbio, Beijing, China; Cat. No. BC0025) according to the manufacturer’s instructions. Briefly, 0.1 g of cherry tomato samples from each treatment group was frozen in liquid nitrogen and thoroughly pulverized. Subsequently, 1 mL of extraction solution was added, and the samples were thoroughly homogenized in an ice bath. The homogenates were centrifuged, and the resulting supernatants were collected for subsequent analysis according to the manufacturer’s instructions. The reaction mixtures of the sample and blank tubes were incubated in a water bath at 100 °C for 60 min and then immediately cooled in an ice bath. The mixtures were subsequently centrifuged at 10,000 r/min for 10 min. An aliquot of 200 μL of the resulting supernatant was transferred to a 96-well microplate, and the absorbance of each sample was measured at 532 and 600 nm. The MDA content was calculated according to the instructions provided with the assay kit [49].

### 2.6. Statistical Analysis

Data were expressed as mean ± SD and analyzed using the GraphPad Prism 8.0.2 statistical software program. Statistical significance was determined using Duncan’s multiple range tests. All experiments were repeated three times. A statistically significant difference between two groups was defined as *p* < 0.05. Different lowercase letters indicated significant differences.

## 3. Results

### 3.1. Antagonistic and Antifungal Activities of Trichoderma asperellum ZZY Against Postharvest Pathogens

The antagonistic activity of *Trichoderma asperellum* ZZY against three postharvest fungal pathogens, *Botrytis cinerea*, *Alternaria alternata*, and *Fusarium oxysporum*, was first evaluated using complementary in vitro assays, including the confrontation assay, cellophane overlay assay, and sandwich plate assay. The inhibition rates obtained from these assays are summarized in Table 1.

**Figure 1:**
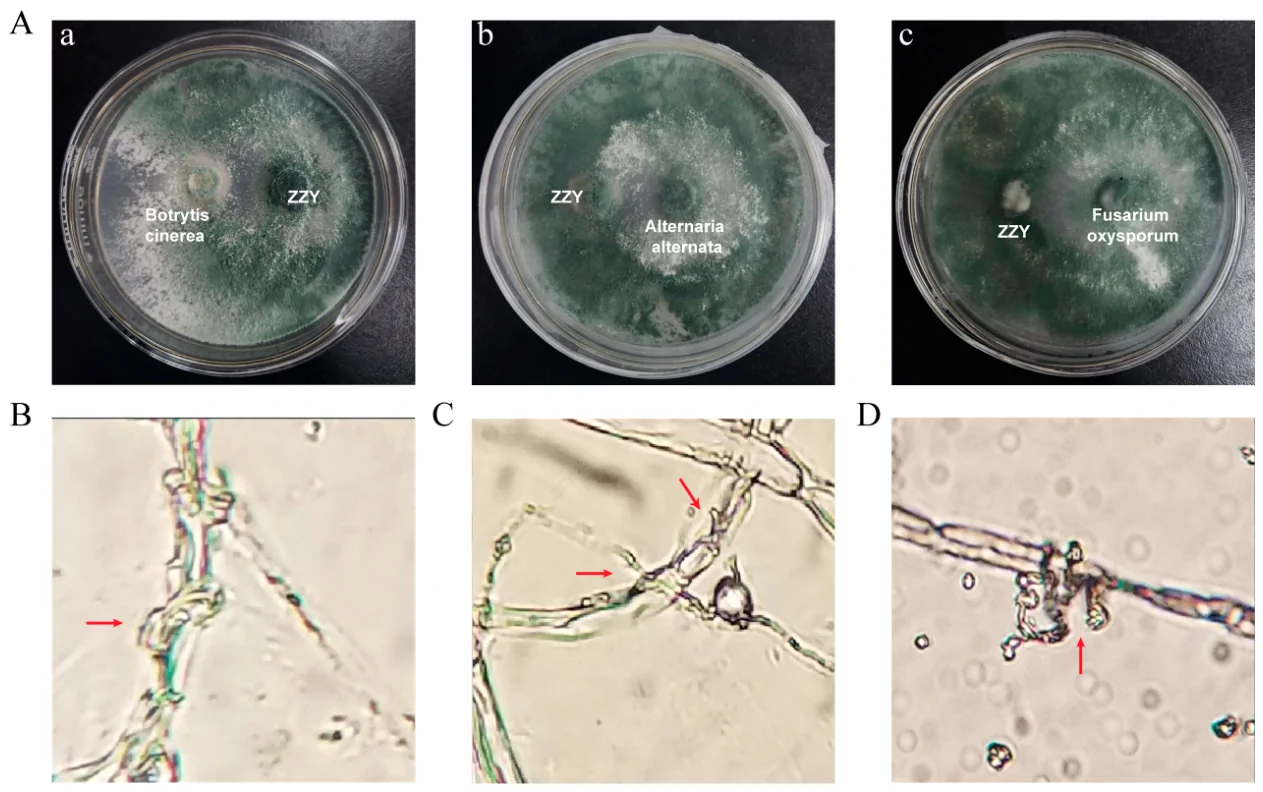
Antagonistic effects of ZZY and fermentation broth on pathogenic fungi. (**A**) Antagonistic effect of ZZY co-cultured with three pathogenic fungi for 5 days ((**a**): *Botrytis cinerea*. (**b**): *Alternaria alternata*. (**c**): *Fusarium oxysporum*). (**B**) Mode of action of ZZY on *Botrytis cinerea* (×400). (**C**) Mode of action of ZZY on *Alternaria alternata* (×400). (**D**) Mode of action of ZZY on *Fusarium oxysporum* (×400). Red arrows indicate the regions of inhibited mycelial growth of the pathogenic fungi caused by *Trichoderma asperellum* ZZY.

In the confrontation assay, ZZY exhibited clear inhibitory effects against all three tested pathogens. The inhibition rates were 67.50% for *Fusarium oxysporum*, 58.33% for *Botrytis cinerea*, and 50.34% for *Alternaria alternat*. These results demonstrated that ZZY effectively inhibited the radial growth of the tested pathogens when directly co-cultured on PDA plates. After 5 days of incubation, the pathogen colonies in the confrontation treatments were visibly smaller than those in the corresponding pathogen-only controls as shown in Figure 1A. Microscopic observations further revealed direct interactions between ZZY hyphae and pathogen hyphae. In particular, ZZY hyphae were observed to entangle and wrap around the pathogen hyphae, which were accompanied by localized constriction and morphological abnormalities at the sites of interaction as shown in Figure 1B–D. These observations suggest that direct hyphal interaction and physical interference may contribute to the antagonistic activity of ZZY against the tested pathogens.

The contribution of non-volatile extracellular factors to the antagonistic activity of ZZY was further evaluated using the Cellophane Overlay Assay. ZZY exhibited substantial inhibitory activity against all three pathogens, with inhibition rates of 78.95%, 79.07%, and 63.77% against *Botrytis cinerea*, *Alternaria alternat*, and *Fusarium oxysporum*, as shown in Table 1. Because the ZZY colony was physically separated from the PDA medium by the cellophane membrane during incubation, these results indicate that diffusible non-volatile extracellular components produced by ZZY may contribute to the inhibition of pathogen growth.

In addition, the Sandwich Plate Assay was used to evaluate the inhibitory effects of volatile compounds produced by ZZY. The inhibition rates were 37.68% for *Fusarium oxysporum*, 32.46% for *Botrytis cinerea*, and 23.26% for *Alternaria alternat* as shown in Table 1. Although the inhibitory effects of the volatile fraction were lower than those observed in the Cellophane Overlay Assay and confrontation assay, the results demonstrate that ZZY also produces volatile compounds capable of inhibiting the growth of the tested pathogens.

Taken together, the results of the three complementary assays demonstrate that ZZY exhibited broad-spectrum antagonistic activity against *Botrytis cinerea*, *Alternaria alternat*, and *Fusarium oxysporum*. The inhibitory effects observed in the confrontation assay, together with those detected in the Cellophane Overlay and Sandwich Plate Assays, suggest that the antagonistic activity of ZZY may involve multiple mechanisms, including direct hyphal interaction and physical interference, as well as the production of diffusible non-volatile and volatile antifungal components.

### 3.2. Antifungal Activity and Stability of ZZY Fermentation Filtrate

To further evaluate the antifungal activity and stability of the ZZY fermentation filtrate, its inhibitory activity against *Botrytis cinerea*, *Alternaria alternata*, and *Fusarium oxysporum* was first monitored during fermentation and subsequently evaluated under different physicochemical treatments, including temperature treatment, storage, UV irradiation, oxidizing and reducing agents, and metal ions.

**Figure 2:**
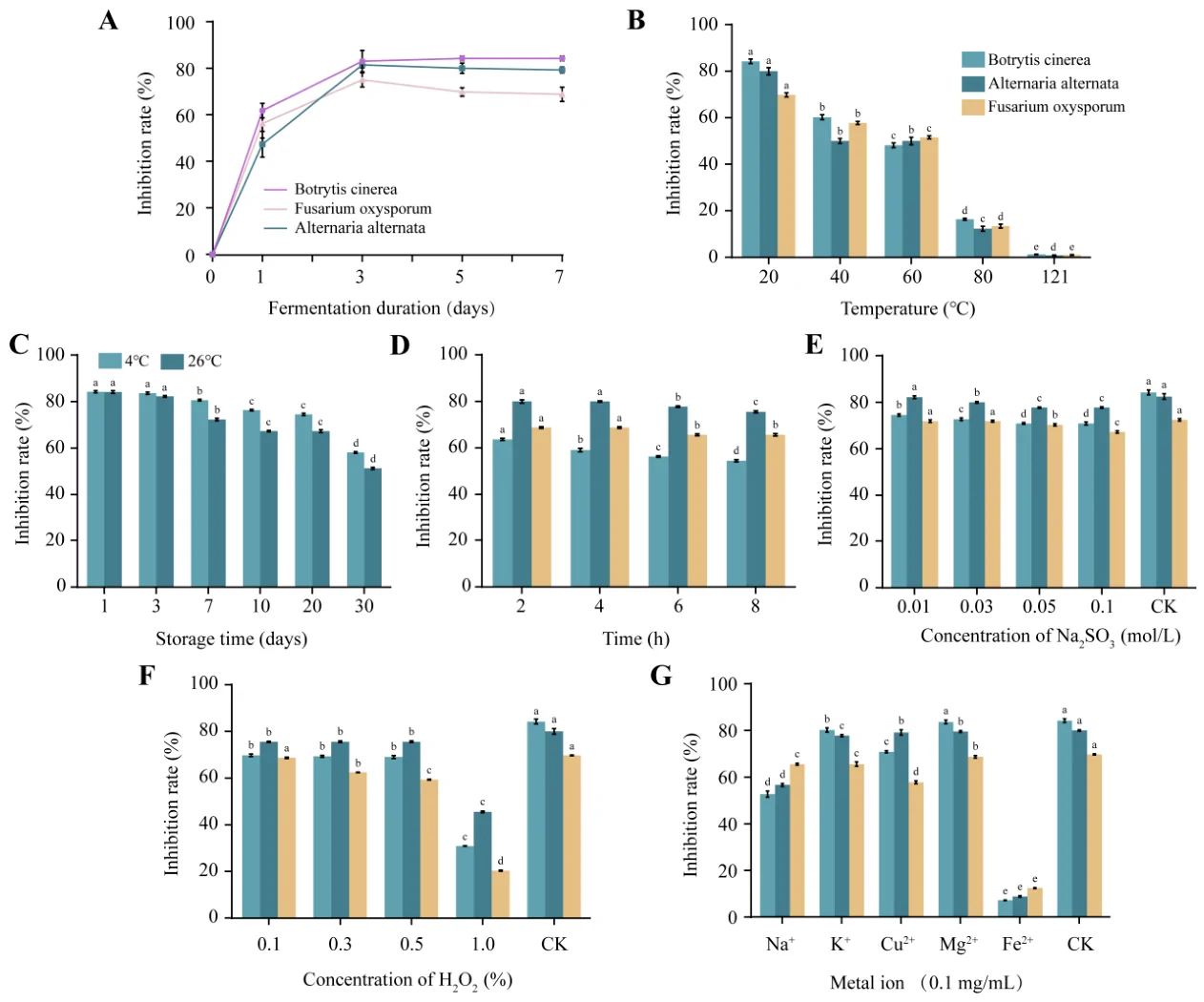
Antifungal stability of ZZY fermentation broth. (**A**) Inhibition rates of ZZY fermentation broth against pathogenic fungi at different fermentation times (*n* = 3). (**B**) Effects of different storage temperatures on the antifungal activity of ZZY fermentation broth (*n* = 3). (**C**) Effects of different storage durations on the stability of antifungal activity in ZZY fermentation broth (*n* = 3). (**D**) Effects of UV treatment on the antifungal activity of ZZY fermentation broth (*n* = 3). (**E**,**F**) Effects of reducing and oxidizing agents on the antifungal activity of ZZY fermentation broth ((**E**) reducing agent; (**F**) oxidizing agent) (*n* = 3). (**G**) Effects of metal ions on the antifungal activity of ZZY fermentation broth (*n* = 3). Different letters in the figure indicate significant differences (*p* < 0.05).

The antifungal activity of the ZZY fermentation filtrate varied with fermentation time as shown in Figure 2A. During the early stages of fermentation, the filtrate showed relatively weak or undetectable inhibitory activity against the three tested pathogens. As fermentation proceeded, the antifungal activity gradually increased. The highest inhibition rates were observed on day 5, reaching 84.24 ± 1.05% against *Botrytis cinerea*, 80.01 ± 2.22% against *Alternaria alternata*, and 69.79 ± 1.81% against *Fusarium oxysporum*. These results indicate that the antifungal activity of the fermentation filtrate increased with fermentation time and reached a maximum after 5 days of fermentation, possibly owing to the accumulation or secretion of extracellular antifungal components during fungal growth.

The thermal stability of the antifungal activity was subsequently evaluated as shown in Figure 2B. The inhibitory activity of the fermentation filtrate against all three pathogens gradually decreased with increasing treatment temperature. At 80 °C, the inhibition rates decreased sharply to below 20%, and almost no antifungal activity remained after treatment at 121 °C. These results indicate that the major antifungal components in the fermentation filtrate were sensitive to high-temperature treatment and may possess relatively poor thermal stability.

The storage stability of the fermentation filtrate was evaluated at 4 °C and 26 °C for different storage periods as shown in Figure 2C. The antifungal activity gradually decreased with increasing storage time at both temperatures. Moreover, the inhibitory activity decreased more rapidly at 26 °C than at 4 °C. These results indicate that storage temperature affected the stability of the antifungal activity, with lower-temperature storage being more favorable for maintaining the activity of the fermentation filtrate.

The effect of UV irradiation on the antifungal activity of the fermentation filtrate was also investigated as shown in Figure 2D. The inhibition rates showed only a slight decrease with increasing UV irradiation time, indicating that the antifungal activity of the fermentation filtrate was relatively stable under the tested UV irradiation conditions. Compared with the pronounced loss of activity observed following high-temperature treatment, UV irradiation had a relatively limited effect on the antifungal activity of the fermentation filtrate.

The effects of oxidizing and reducing agents were further examined as shown in Figure 2E,F. Increasing concentrations of sodium sulfite (Na_2_SO_3_) resulted in a slight decrease in antifungal activity. In contrast, treatment with hydrogen peroxide (H_2_O_2_), particularly at 1.00%, caused a pronounced reduction in the inhibition rate. These results suggest that the antifungal activity of the fermentation filtrate was more sensitive to oxidative treatment than to the tested reducing conditions, indicating that oxidation-sensitive components may contribute to its antifungal activity.

Finally, the effects of different metal ions on the antifungal activity of the fermentation filtrate were evaluated as shown in Figure 2G. Compared with the untreated control, the addition of Fe^2+^ markedly reduced the inhibition rate to approximately 10%. Na^+^, K^+^, and Cu^2+^ caused relatively minor decreases in antifungal activity, whereas the addition of Mg^2+^ had no significant effect under the tested conditions. These results indicate that the antifungal activity of the fermentation filtrate was relatively stable in the presence of Mg^2+^, Na^+^, K^+^, and Cu^2+^, whereas Fe^2+^ had a pronounced inhibitory effect on the activity.

Overall, the antifungal activity of the ZZY fermentation filtrate was strongly affected by fermentation time, temperature, storage conditions, oxidative treatment, and certain metal ions, whereas relatively limited changes were observed following UV irradiation, reducing treatment, and exposure to Mg^2+^, Na^+^, K^+^, and Cu^2+^ ions. These findings provide preliminary information regarding the physicochemical stability of the antifungal activity of the ZZY fermentation filtrate and provide a basis for subsequent investigation of its active components.

### 3.3. Isolation, Purification, and Characterization of the Antifungal Protein TAFLP from Trichoderma asperellum ZZY Fermentation Filtrate

The ZZY fermentation filtrate exhibited substantial antifungal activity against the three tested pathogens. To further identify the types and physicochemical characteristics of the active components, a series of fractionation, compositional, and biochemical analyses was performed.

#### 3.3.1. Preliminary Fractionation and Analysis of Active Components

The ZZY fermentation filtrate was sequentially extracted using solvents with increasing polarity. As shown in Table 2, the petroleum ether, ethyl acetate, and n-butanol fractions exhibited relatively weak inhibitory activity against *Botrytis cinerea*, *Alternaria alternata*, and *Fusarium oxysporum*. In contrast, the residual aqueous fraction retained substantially higher antifungal activity, with inhibition rates of 68.97%, 80.00%, and 69.70% against *Botrytis cinerea*, *Alternaria alternata*, and *Fusarium oxysporum*, respectively. These results indicate that the major antifungal activity was preferentially retained in the aqueous fraction, suggesting that relatively hydrophilic components may contribute substantially to the antifungal activity of the ZZY fermentation filtrate.

**Figure 3:**
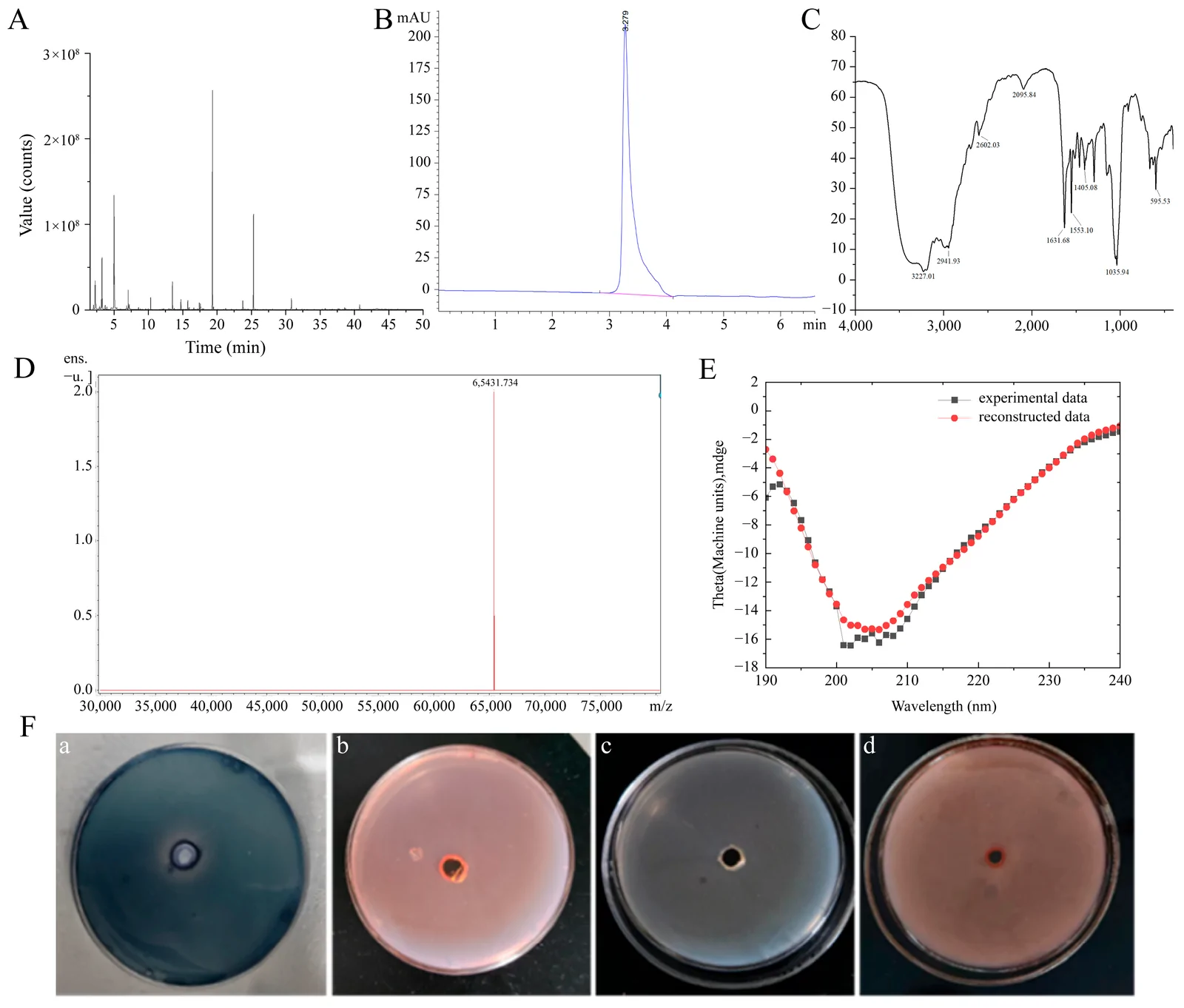
Analysis of Antifungal Compounds in the Fermentation Filtrate of ZZY. (**A**) GC–MS chromatogram of volatile compounds in ZZY fermentation broth. (**B**) HPLC chromatogram of TAFLP (The blue line represents the chromatographic peak, and the pink line indicates the integrated peak area.). (**C**) FT-IR spectrum of TAFLP. (**D**) MALDI-TOF mass spectrum of TAFLP. (**E**) CD spectrum of TAFLP. (**F**) Enzymatic activity assay of the antifungal protein ((**a**): chitinase. (**b**): β-1,3-glucanase. (**c**): protease. (**d**): cellulase).

The volatile components of the ZZY fermentation broth were subsequently analyzed by HS-SPME-GC-MS. A total of 71 volatile compounds were detected, with alcohols and alkanes accounting for 54.02% and 20.49% of the total detected volatile components, as shown in Figure 3A. Other compounds, including palmitic acid, nonanal, and 3-hydroxy-2-butanone, were also detected at relatively lower abundances in Supplementary Table S1. The presence of these diverse volatile compounds indicates that volatile metabolites may contribute to the overall antifungal activity of the ZZY fermentation products. However, because the antifungal activities of the individual compounds were not independently evaluated in the present study, their specific contributions to the observed antifungal activity remain to be determined.

To further investigate the potential contribution of macromolecular components, crude protein and crude polysaccharide fractions were prepared from the ZZY fermentation broth and their antifungal activities were evaluated. As shown in Table 3, the crude protein fraction exhibited inhibition rates of approximately 50–60% against the three tested pathogens. In comparison, the crude polysaccharide fraction showed substantially weaker inhibition against *Botrytis cinerea* and *Alternaria alternata*, and no detectable inhibition against *Fusarium oxysporum*. These results suggest that proteinaceous components may represent important contributors to the antifungal activity of the ZZY fermentation broth.

#### 3.3.2. Purification and Structural Characterization of TAFLP

Based on the relatively strong antifungal activity observed for the crude protein fraction, further purification was performed using Sephadex G-100 gel filtration chromatography as shown in Supplementary Figure S1. The purified protein fraction exhibited a single major peak at a retention time of 3.729 min in the subsequent HPLC analysis as shown in Figure 3B and was designated as TAFLP.

The chemical and structural characteristics of TAFLP were subsequently investigated using FTIR spectroscopy, MALDI-TOF MS, and CD spectroscopy. The FTIR spectrum of TAFLP showed several characteristic absorption bands associated with protein structures as shown in Figure 3C. The absorption band at 1631.68 cm^−1^ was mainly assigned to the amide I region, whereas the band at 1553.10 cm^−1^ was associated with the amide II region. The broad absorption band near 3227 cm^−1^ was attributable mainly to O-H and N-H stretching vibrations. The absorption band near 2942 cm^−1^ was associated with aliphatic C-H stretching vibrations. Additional absorption bands were observed at approximately 1403 and 1037 cm^−1^, which may be related to C-H bending and C-O-containing group vibrations, respectively. Overall, the FTIR profile provided evidence supporting the proteinaceous characteristics of TAFLP.

MALDI-TOF MS analysis estimated the molecular mass of TAFLP to be approximately 6.54 × 10^4^ Da as shown in Figure 3D. CD spectroscopy was further employed to characterize the secondary structure of TAFLP as shown in Figure 3E. The CD spectrum in the far-UV region (190–240 nm) reflected the conformational characteristics of the peptide backbone. According to the DichroWeb analysis, TAFLP contained α-helical, β-sheet, turn, and random-coil structural elements as shown in Table 4, indicating that the purified protein possessed a defined secondary structure.

#### 3.3.3. Enzymatic Activity of TAFLP

Because the structural characterization indicated that TAFLP was a proteinaceous component with substantial antifungal activity, its potential enzymatic activities were further investigated as shown in Figure 3F. TAFLP produced a clear hydrolysis zone on the colloidal chitin plate, indicating detectable chitinase activity. In contrast, no visible hydrolysis zones were observed on the laminarin, skim milk, or CMC-Na plates, suggesting that β-1,3-glucanase, protease, and cellulase activities were not detected under the experimental conditions used in this study.

Taken together, the fractionation and biochemical analyses indicate that the antifungal activity of the ZZY fermentation filtrate is associated with multiple extracellular components. The relatively high activity retained in the aqueous fraction, together with the stronger activity of the crude protein fraction, supports the potential contribution of hydrophilic proteinaceous components. Further purification and characterization identified TAFLP as a proteinaceous component with a molecular mass of approximately 6.54 × 10^4^ Da and detectable chitinase activity. These findings suggest that TAFLP may contribute to the antifungal activity of the ZZY fermentation products, although its specific contribution relative to other volatile and non-volatile components requires further investigation.

### 3.4. Optimization of Fermentation Conditions for Antifungal Protein Production by Trichoderma asperellum ZZY

The fermentation broth serves as a microenvironment for fungal growth, in which each component functions as a regulatory factor that directly or indirectly influences protein production and secretion. The composition and physicochemical conditions of the ZZY fermentation broth may affect the production of antifungal proteins. To determine the optimal carbon source, six readily available carbon sources, namely glucose, starch, lactose, sucrose, maltose, and a combination of glucose and starch, were added to the culture medium. As shown in Table 5, the different carbon sources significantly affected the protein content of the fermentation broth. As shown in Table 6, when lactose was used as the carbon source at a concentration of 4.0%, the protein content reached a maximum of 1.48 mg/mL, which was markedly higher than that obtained in the original PDB medium (0.25 mg/mL). Therefore, 4% lactose was selected as the optimal carbon source.

Nitrogen is essential for microbial growth and fermentation. Readily available nitrogen sources can be rapidly utilized by microorganisms and promote biomass accumulation, whereas slowly available nitrogen sources may prolong the metabolite secretion phase and increase product yield. However, excessive nitrogen supplementation at the beginning of fermentation may accelerate microbial growth, resulting in premature nutrient depletion, cellular aging, and autolysis, thereby shortening the product secretion period. To investigate the effects of different nitrogen sources, six nitrogen treatments comprising organic, inorganic, and mixed nitrogen sources were evaluated: yeast extract, peptone, (NH_4_)_2_SO_4_, soybean meal powder, peptone + (NH_4_)_2_SO_4_, and yeast extract + (NH_4_)_2_SO_4_. As shown in Table 7, the protein content of the fermentation broth differed significantly among the different nitrogen sources. The combination of peptone and (NH_4_)_2_SO_4_ at a total concentration of 1% resulted in the highest protein content (1.87 mg/mL), as shown in Table 8. Therefore, the combination of peptone and (NH_4_)_2_SO_4_ at a total concentration of 1% was selected as the optimal nitrogen source.

The initial pH of the culture medium also affected the protein content of the fermentation broth. As shown in Table 9, when the initial pH was adjusted to 5.73, 6.0, 7.0, 8.0, and 9.0, the protein content reached a maximum of 1.80 mg/mL at pH 7.0. Both excessively acidic and alkaline conditions inhibited protein production. Therefore, an initial pH of 7.0 was selected as the optimal condition.

The effect of fermentation duration (2, 3, 4, 5, and 6 days) on protein content was further investigated. As shown in Table 10, the protein content initially increased and subsequently decreased with increasing fermentation time, reaching a maximum of 1.78 mg/mL on day 4. During the early stage of fermentation, ZZY may not have fully utilized the available nutrients, resulting in relatively low protein production. As fermentation progressed, nutrient utilization increased, leading to an accumulation of extracellular proteins and a peak in protein content. However, prolonged fermentation resulted in a decrease in protein content, possibly due to nutrient depletion and subsequent degradation or utilization of extracellular proteins. Therefore, the optimal fermentation duration was determined to be 4 days.

The filling volume also significantly affected protein production. As shown in Table 11, the protein content increased with increasing filling volume and subsequently decreased after reaching a maximum of 1.72 mg/mL at a filling volume of 120 mL. Therefore, a filling volume of 120 mL was selected as optimal.

Based on these results, the optimized fermentation conditions for ZZY were determined as follows: 4% lactose as the carbon source, a combination of peptone and (NH_4_)_2_SO_4_ at a total concentration of 1% as the nitrogen source, an initial pH of 7.0, a fermentation duration of 4 days, and a filling volume of 120 mL.

### 3.5. Effect of Trichoderma asperellum ZZY Fermentation Filtrate on the Postharvest Quality and Physiological Changes in Cherry Tomatoes

The preservation effect of ZZY fermentation broth on cherry tomatoes was evaluated during storage by monitoring changes in firmness, weight loss, decay rate, color, soluble solids content, TA, and MDA content. These parameters were selected to comprehensively assess the effects of the treatment on fruit texture, water loss, decay development, physicochemical quality, and membrane lipid peroxidation.

**Figure 4:**
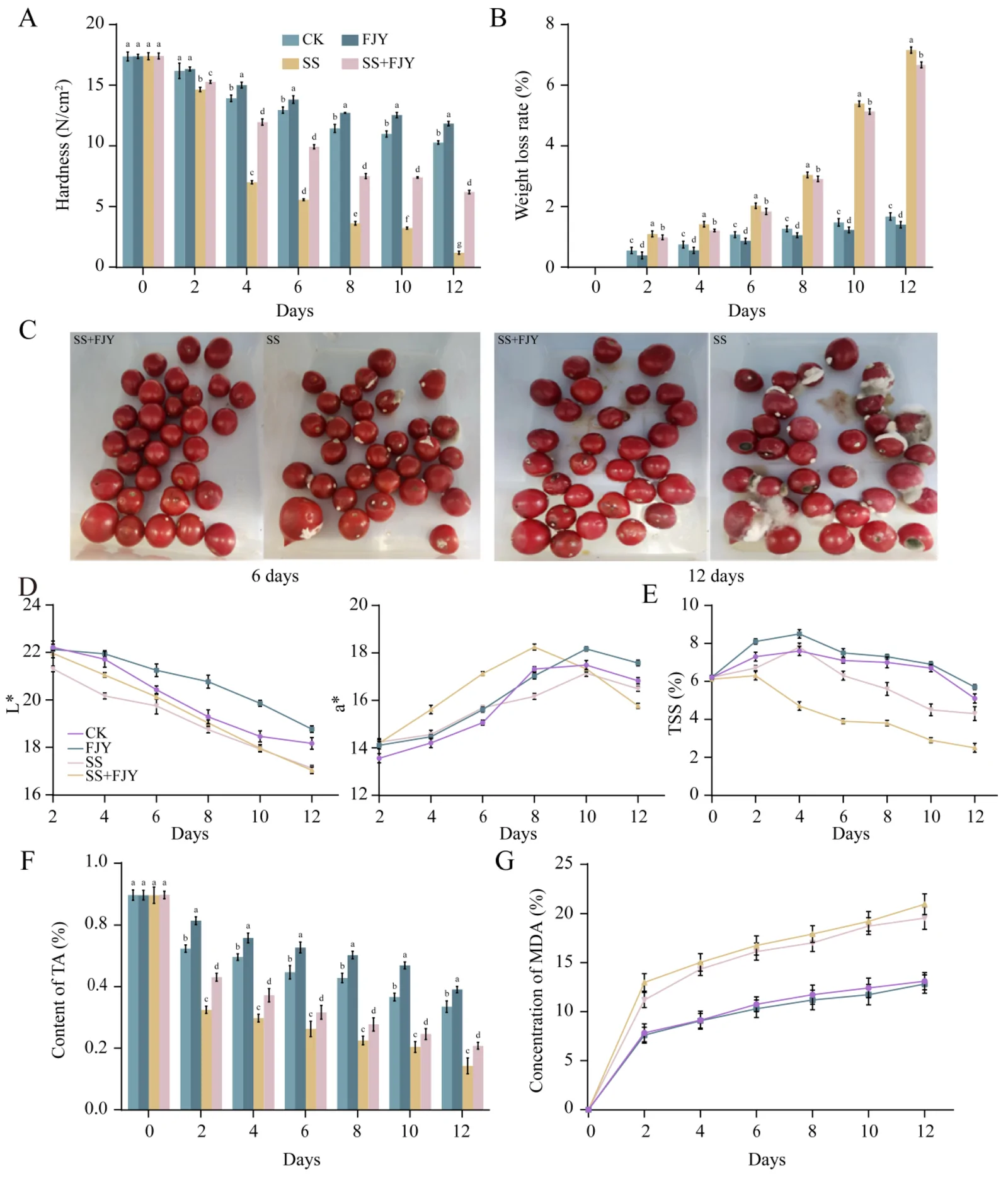
Evaluation of the preservation effect of ZZY fermentation liquid on cherry tomatoes. (**A**) Changes in fruit firmness of cherry tomatoes under different treatments (*n* = 3). (**B**) Effects of different treatments on the weight loss rate of cherry tomatoes (*n* = 3). (**C**) Degree of fruit decay in cherry tomatoes on days 6 and 12. (**D**) Changes in surface color parameters (L* and a*) of cherry tomatoes during storage (a: changes in L* value. b: changes in a* value) (*n* = 3). (**E**) Changes in soluble solids content of cherry tomatoes during storage (*n* = 3). (**F**) Changes in titratable acidity of cherry tomatoes during storage (*n* = 3). (**G**) Changes in malondialdehyde (MDA) content of cherry tomatoes during storage (*n* = 3). Different letters in the figure indicate significant differences (*p* < 0.05).

During fruit ripening and post-harvest storage, pectin is degraded by pectinases into pectic acids, leading to cell separation and disintegration, a decline in fruit firmness, and overall deterioration of fruit quality during storage [50,51]. Fruit firmness decreased progressively during storage in all treatment groups as shown in Figure 4A. In the CK group, firmness decreased by 7.10 N/cm^2^ by day 12. Firmness in the SS group began to decline gradually from day 2 and reached 1.20 N/cm^2^ on day 12, corresponding to a 93.09% reduction relative to the initial value. In the FJY group, firmness decreased by 5.55 N/cm^2^ by day 12, and the final firmness was 21.77% higher than that of the SS group. The SS+FJY group maintained a firmness of 6.20 N/cm^2^ on day 12, corresponding to a decrease of 11.21 N/cm^2^ from the initial value and a value 5.01 N/cm^2^ higher than that of the SS group. These results indicate that treatment with ZZY fermentation broth helped maintain fruit firmness, particularly in inoculated fruit. Fruit weight loss increased gradually with storage time in all groups, which is consistent with the progressive loss of water associated with respiration and evaporation during storage. As shown in Figure 4B, on day 12, the weight loss rates of the CK and FJY groups were 1.67% and 1.41%, respectively. In comparison, the SS group exhibited a substantially higher weight loss rate of 7.16%, whereas the SS+FJY group showed a slightly lower value of 6.67%. Thus, the addition of ZZY fermentation broth was associated with a reduction in weight loss in SS-treated fruit, although the difference between SS and SS+FJY was relatively limited. Decay development was also markedly affected by the treatments. As shown in Figure 4C, visible decay became apparent in the SS group by day 6, whereas only slight decay was observed in the FJY group at the same time point. By day 12, extensive mold growth and decay were observed in the SS group, with a decay rate of 54.68%. In contrast, the FJY group showed a lower decay rate of 32.14%. The reduced decay observed following fermentation broth treatment is consistent with the antifungal activity of ZZY observed in the in vitro assays and suggests that the fermentation product may contribute to the suppression of postharvest fungal decay.

Fruit color, soluble solids content, and TA were further monitored to evaluate changes in physicochemical quality during storage (Figure 4D–F). Brightness gradually decreased in all groups during storage as shown in Figure 4D. By day 12, the decreases in brightness were 2.48, 3.36, 1.88, and 3.00 units in the CK, SS, FJY, and SS+FJY groups, respectively. The red-green hue increased initially and subsequently decreased during storage. In the SS group, the hue value reached a maximum on day 8 and then declined. The decline was slower in the SS+FJY group than in the SS group, while the FJY group showed a slower decline than the CK group. These results indicate that the treatments affected the progression of color changes during storage, although the responses differed among the individual color parameters. The soluble solids content gradually increased during the early stage of storage and began to decline after day 3 in all groups as shown in Figure 4E. Compared with the SS group, the FJY group exhibited a slower decline in soluble solids content during the later storage period, suggesting that fermentation broth treatment may have helped delay the deterioration of soluble-solids-related quality during storage. TA decreased progressively throughout storage in all groups, with differences observed among treatments as shown in Figure 4F. By day 12, TA had decreased by 0.36%, 0.31%, 0.55%, and 0.49% in the CK, FJY, SS, and SS+FJY groups, respectively. The decrease in the SS+FJY group was 0.06% lower than that in the SS group, indicating that fermentation broth treatment partially alleviated the decline in TA in inoculated fruit.

Under adverse conditions, free radicals generated in fruit and vegetable cells trigger lipid peroxidation, increasing membrane permeability, damaging cell structure and function, and reducing nutritional value [52]. MDA content was measured as an indicator associated with membrane lipid peroxidation during storage. As shown in Figure 4G, MDA content increased progressively in all groups. On day 12, the MDA contents of the CK and FJY groups were 13.11 and 12.82 nmol/g, respectively, with the latter being 0.29 nmol/g lower than the CK group. In the SS group, MDA content increased to 20.97 nmol/g, whereas the SS+FJY group showed a lower value of 19.55 nmol/g, representing a decrease of 1.42 nmol/g relative to the SS group. The lower MDA accumulation in the SS+FJY group suggests that ZZY fermentation broth treatment was associated with reduced membrane lipid peroxidation in inoculated cherry tomatoes during storage. Overall, ZZY fermentation broth treatment was associated with reduced decay, lower weight loss, better retention of firmness, and lower MDA accumulation in inoculated cherry tomatoes, together with relatively slower changes in several physicochemical quality parameters. These results demonstrate that the ZZY fermentation product can delay quality deterioration and suppress postharvest decay under the conditions tested, supporting its potential application as a biological preservation treatment for cherry tomatoes.

## 4. Discussion

Postharvest fungal decay is an important factor limiting the quality and shelf life of fresh fruits and vegetables. *Trichoderma asperellum* has been investigated as a biological control agent against plant pathogens, with reported protective effects involving both direct antagonism and host-mediated defense responses [53]. Herrera-Téllez et al. [54] investigated the protective effect of *Trichoderma asperellum* against tomato diseases caused by *Fusarium oxysporum* and *Botrytis cinerea*, highlighting the involvement of reactive oxygen species-related responses. Similarly, Wang et al. [55] reported that *Trichoderma asperellum* strain DQ-1 inhibited *Botrytis cinerea* and induced resistance to gray mold in tomato plants. The inhibition rate of DQ-1 against *Botrytis cinerea* was 88.56% under their experimental conditions, and treatment reduced tomato disease incidence and severity. These studies support the potential of *Trichoderma asperellum* for managing tomato fungal diseases, while also demonstrating that its effects may involve mechanisms beyond direct pathogen inhibition. In the present study, *Trichoderma asperellum* ZZY inhibited *Botrytis cinerea*, *Alternaria alternata*, and *Fusarium oxysporum*, with inhibition rates of 58.33%, 50.34%, and 67.50%, respectively, in the confrontation assay. The different inhibition rates observed in the present study and those reported for strain DQ-1 should not be interpreted as evidence of inferior or superior biocontrol potential because the fungal strains, experimental systems, and evaluation conditions differed. Moreover, the present study focused primarily on the antifungal properties of fermentation products rather than on the induction of host resistance. The results therefore complement previous research by providing evidence that ZZY-associated antagonism may involve extracellular antifungal factors, although their individual contributions require further investigation.

The antifungal activity of ZZY involved both volatile and non-volatile components. The volatile fraction inhibited *Fusarium oxysporum*, *Botrytis cinerea*, and *Alternaria alternata* by 37.68%, 32.46%, and 23.26%, respectively. HS-SPME-GC-MS analysis identified 71 volatile compounds, predominantly alcohols (54.02%) and alkanes (20.49%). However, the non-volatile fraction exhibited stronger antifungal activity, with inhibition rates of 78.95%, 79.07%, and 63.77% against the three pathogens, respectively. Furthermore, the aqueous fraction retained considerable activity, suggesting that hydrophilic extracellular components, potentially including proteins and other water-soluble metabolites, contribute substantially to the antifungal effects. Nevertheless, the specific active compounds and their individual contributions remain to be determined through further chemical and biochemical characterization.

Further analyses showed that TAFLP exhibited typical protein characteristics and possessed chitin-hydrolyzing activity. Previous research has established that *Trichoderma asperellum* can produce chitinases with measurable biological activities. Berini et al. [56] characterized chitinases produced by *Trichoderma asperellum* ICC012 and demonstrated their effects on the chitin microfibril network of the insect peritrophic matrix, including enhanced effects when used in combination with Bacillus thuringiensis. Although this study concerned insect control rather than fungal pathogens, it provides evidence that *Trichoderma asperellum*-derived chitinases can act on chitin-containing structures. Liu et al. [57] further demonstrated that chitinase production in *Trichoderma asperellum* T4 is associated with the regulation of N,N′-diacetylchitobiose transport, highlighting the biological regulation of chitin utilization and chitinase production in this species. These studies provide a relevant biochemical context for the chitin-hydrolyzing activity detected in TAFLP. Because chitin is an important structural component of the cell walls of most fungi, chitin-degrading enzymes could potentially weaken pathogen cell walls and interfere with hyphal growth. Nevertheless, the findings do not directly demonstrate that the chitin-hydrolyzing activity of ZZY fermentation products is responsible for the inhibition of the three pathogens examined here. The present results support the possibility that TAFLP contributes to antifungal activity, but they do not establish it as the sole or determinant active component. The stronger activity of the complete fermentation filtrate relative to some individual fractions also suggests that other extracellular proteins or soluble metabolites may contribute to the overall effect. Whether these components act additively or synergistically remains unresolved. Additional purification, protein inactivation or inhibition assays, activity-recovery experiments, and direct assessment of pathogen cell wall integrity are therefore necessary to determine the specific contribution of TAFLP and clarify its interaction with other active components.

The improvement in cherry tomato storage quality following treatment with ZZY fermentation products supports their potential application in postharvest disease management. Previous studies have demonstrated that *Trichoderma asperellum*-derived metabolites can contribute to fungal inhibition and fruit protection. For example, Alfaro-Vargas et al. [58] characterized peptaibols produced by *Trichoderma asperellum* and reported inhibition rates of 74.2%, 58.4%, and 36.2% against *Botrytis cinerea*, *Alternaria alternata*, and *Fusarium oxysporum*, respectively; their peptaibol extract also reduced *A. alternata* infection in inoculated tomatoes. These findings provide a relevant comparison with the present study, in which ZZY fermentation products exhibited antifungal activity against the same three pathogens and improved cherry tomato preservation. Nevertheless, the inhibition rates cannot be directly compared because the composition and concentration of the tested preparations, as well as the experimental conditions, differed between studies. Recent reviews have further highlighted the potential of *Trichoderma* species for postharvest disease control while emphasizing the need to clarify their mechanisms of action, formulation stability, and practical applicability [59,60]. In the present study, the preservation effect may be associated with the combined activity of extracellular proteins, hydrophilic metabolites, and volatile compounds, although their individual contributions and interactions remain to be established. Fermentation-derived preparations may offer advantages over live-microorganism formulations by facilitating control of product composition and application dosage; however, these advantages require direct comparative validation. In addition, although peptaibols have been identified in some *Trichoderma* strains [61], their presence in ZZY fermentation products was not determined in this study and should not be assumed. Further investigations should therefore evaluate the stability and batch-to-batch consistency of the active components, identify potentially harmful secondary metabolites, assess cytotoxicity and allergenic potential, and quantify residual fungal biomass and viable spores. Such studies, together with comparisons against established postharvest treatments and evaluations under practical storage conditions, will be essential for establishing the efficacy, safety, and commercial feasibility of ZZY fermentation products as potential postharvest biocontrol agents.

Overall, this study demonstrates that ZZY and its fermentation products exhibit inhibitory activity against multiple postharvest fungal pathogens of cherry tomatoes. In the context of previous research on *Trichoderma asperellum*, the findings complement reports of plant disease suppression and induced resistance, chitinase production and biological activity, and antifungal peptaibols. The results suggest that the antifungal activity of ZZY may involve multiple extracellular factors, including volatile metabolites, hydrophilic components, and proteinaceous material with chitin-hydrolyzing activity. TAFLP may represent an important contributor to this activity, although its specific contribution and interactions with other components remain to be established. The observed preservation effects further support the need to investigate ZZY fermentation products as potential postharvest biocontrol preparations. Further comparative studies, activity-guided purification, mechanistic validation, and systematic food-safety assessments are required to establish their reproducibility, mode of action, and practical applicability.

## 5. Conclusions

Overall, the present study demonstrated that *Trichoderma asperellum* ZZY possesses multiple complementary antifungal mechanisms and has potential as a biological resource for postharvest disease management of cherry tomatoes. In the confrontation assay, ZZY inhibited the growth of *Fusarium oxysporum*, *Botrytis cinerea*, and *Alternaria alternata* by 67.50%, 58.33%, and 50.34%, respectively, accompanied by direct antagonistic interactions such as hyphal coiling and constriction. Volatile and non-volatile assays further demonstrated that both volatile metabolites and diffusible extracellular components contributed to the overall antifungal activity. The ZZY fermentation broth also showed substantial inhibitory activity against the three pathogens, with maximum inhibition rates of 84.24%, 80.01%, and 69.79% against *Botrytis cinerea*, *Alternaria alternata*, and *Fusarium oxysporum*, respectively. Solvent fractionation indicated that the antifungal activity was predominantly retained in the aqueous fraction, suggesting an important contribution of hydrophilic extracellular components. Further characterization of the proteinaceous component, TAFLP, together with enzymatic assays, revealed chitinase activity, suggesting that disruption of pathogen cell-wall integrity may contribute to its antifungal effect. Importantly, application of the fermentation broth to cherry tomatoes reduced decay and weight loss while helping maintain fruit firmness and physicochemical quality during storage. These findings establish a link between the multiple antagonistic mechanisms of ZZY, its fermentation-derived antifungal components, and postharvest disease suppression, providing a scientific basis for the further development of fermentation-derived biological preservatives. Nevertheless, further studies are required to clarify the molecular identity and mode of action of TAFLP, evaluate the interactions among different antifungal components, and systematically assess its stability, safety, and formulation characteristics before practical application.

## Figures and Tables

**Table 1:** The inhibitory effects of ZZY on pathogenic fungi.

Pathogenic Fungi	Rate of Inhibition (%)		
	*Botrytis cinerea*	*Alternaria alternata*	*Fusarium oxysporum*
Inhibition of plate	58.33 ± 1.03	50.34 ± 3.12	67.50 ± 5.00
Non-volatile substance	78.95 ± 1.21	79.07 ± 0.87	63.77 ± 2.51
Volatile substance	32.46 ± 3.46	23.26 ± 2.33	37.68 ± 2.15

**Table 2:** Antimicrobial activity of each phase of *Trichoderma asperellum* ZZY fermentation broth.

Phase of Extraction	Rate of Inhibition (%)		
	*Botrytis cinerea*	*Alternaria alternata*	*Fusarium oxysporum*
N-butanol phase	29.31 ± 1.33 ^a^	36.00 ± 2.01 ^a^	24.24 ± 1.54 ^a^
Ethyl acetate phase	12.07 ± 2.01 ^b^	26.00 ± 1.33 ^b^	18.18 ± 1.77 ^a^
Petroleum ether phase	22.41 ± 1.57 ^a^	32.00 ± 1.71 ^a^	9.09 ± 1.03 ^b^
Water Phase	68.97 ± 1.05 ^c^	80.00 ± 2.22 ^c^	69.70 ± 1.81 ^c^

Different letters in the table indicate significant differences (*p* < 0.05).

**Table 3:** Inhibition rate of crude protein and crude polysaccharide in the fermentation broth of ZZY on pathogenic fungi.

	Rate of Inhibition (%)		
	*Botrytis cinerea*	*Alternaria alternata*	*Fusarium oxysporum*
Crude protein	58.62 ± 0.43 ^a^	52.00 ± 1.31 ^a^	60.61 ± 1.57 ^a^
Crude polysaccharide	29.31 ± 1.01 ^b^	26.00 ± 0.74 ^b^	1.32 ± 1.03 ^b^
Brothfreeze-dried of fermentation broth	56.90 ± 1.47 ^a^	54.00 ± 0.83 ^a^	48.48 ± 1.32 ^c^
TAFLP (5 mg/mL)	67.24 ± 0.97 ^c^	62.00 ± 1.09 ^c^	66.77 ± 1.24 ^d^

Different letters in the table indicate significant differences (*p* < 0.05).

**Table 4:** Proportion of secondary structure of TAFLP.

	Helix	Strand	Turns	Unordered
TAFLP (%)	22.2	21.7	23	35.1

**Table 5:** The influence of different carbon sources on the protein content of fermentation broth.

Carbon Source	Protein Content of the Fermentation Broth (mg/mL)
Glucose	0.43 ± 0.07 ^b^
Starch	0.46 ± 0.03 ^b^
Lactose	0.67 ± 0.13 ^a^
Sucrose	0.45 ± 0.07 ^b^
Maltose	0.57 ± 0.06 ^ab^
Glucose + Starch	0.39 ± 0.02 ^b^

Different letters in the table indicate significant differences (*p* < 0.05).

**Table 6:** Effect of lactose addition on protein content in fermentation broth.

Lactose Content (%)	Protein Content of the Fermentation Broth (mg/mL)
1.0	0.52 ± 0.01 ^c^
2.0	0.82 ± 0.03 ^bc^
3.0	1.09 ± 0.05 ^ab^
4.0	1.48 ± 0.37 ^a^

Different letters in the table indicate significant differences (*p* < 0.05).

**Table 7:** Effect of different nitrogen sources on protein content in fermentation broth.

Nitrogen Source	Protein Content of the Fermentation Broth (mg/mL)
Yeast extract powder	1.28 ± 0.06 ^bc^
Peptone	1.30 ± 0.05 ^abc^
(NH_4_)_2_SO_4_	1.08 ± 0.07 ^c^
Soybean meal powder	1.41 ± 0.17 ^ab^
Peptone + (NH_4_)_2_SO_4_	1.52 ± 0.03 ^a^
Yeast extract powder +(NH_4_)_2_SO_4_	1.45 ± 0.05 ^ab^

Different letters in the table indicate significant differences (*p* < 0.05).

**Table 8:** Effect of peptone + ammonium sulfate addition on protein content in fermentation broth.

Peptone + (NH_4_)_2_SO_4_ Addition Amount (%)	Protein Content of the Fermentation Broth (mg/mL)
0.1	1.52 ± 0.03 ^d^
0.25	1.52 ± 0.04 ^d^
0.5	1.64 ± 0.03 ^c^
0.75	1.74 ± 0.03 ^b^
1	1.87 ± 0.03 ^a^

Different letters in the table indicate significant differences (*p* < 0.05).

**Table 9:** Effect of initial pH on protein content in fermentation broth.

pH	Protein Content of the Fermentation Broth (mg/mL)
9.0	1.28 ± 0.03 ^c^
8.0	1.77 ± 0.04 ^a^
7.0	1.80 ± 0.07 ^a^
6.0	1.59 ± 0.05 ^b^
nature (5.73)	1.36 ± 0.04 ^c^

Different letters in the table indicate significant differences (*p* < 0.05).

**Table 10:** Effect of fermentation time on protein content of fermentation broth.

Fermentation Time (Days)	Protein Content of the Fermentation Broth (mg/mL)
2	1.54 ± 0.04 ^bc^
3	1.59 ± 0.04 ^b^
4	1.78 ± 0.04 ^a^
5	1.48 ± 0.05 ^bc^
6	1.44 ± 0.05 ^c^

Different letters in the table indicate significant differences (*p* < 0.05).

**Table 11:** Effect of liquid filling on protein content of fermentation broth.

Liquid Filling Volume (mL)	Protein Content of the Fermentation Broth (mg/mL)
150	1.45 ± 0.03 ^c^
120	1.72 ± 0.06 ^a^
100	1.61 ± 0.05 ^b^
80	1.61 ± 0.03 ^ab^
60	1.53 ± 0.03 ^bc^

Different letters in the table indicate significant differences (*p* < 0.05).

## Data Availability

The original contributions presented in the study are included in the article/Supplementary Material; further inquiries can be directed to the corresponding author.

## Supplementary Materials

The following supporting information can be downloaded at: https://www.mdpi.com/article/10.3390/foods15203682/s1. Supplementary Table S1 Table of main volatile substances in the fermentation broth of *Trichoderma asperellum* ZZY. Supplementary Figure S1 Elution curve of crude protein on SepHadexG-100.
