# Supplementary material for: Antifungal Activity of Trichoderma asperellum ZZY Fermentation Products Against Postharvest Cherry Tomato Pathogens and Their Preservation Effect
Source: Foods. 2026 Oct 16;15(20):3682. doi: 10.3390/foods15203682 (PMC13703773; doi:10.3390/foods15203682)
Supplement: Supplementary file 1 [file foods-15-03682-s001.zip › foods-4582786-supplementary.pdf]

Supplementary Table S1 Table of main volatile substances in the fermentation broth of *Trichoderma asperellum* ZZY

Name	Ret.Time	Molecular formula	MW	Relative content (%)
Carbon dioxide	2.01	CO_2	44.009	2.79
Ethanol	2.25	C_2H_6O	46.068	18.25
2,3-Butanedione	2.88	$C_4H_6O_2$	86.089	1.55
Trichloromethane	3.14	$CHCl_3$	119.38	3.61
2-Methyl-1-propanol	3.24	$C_4H_{10}O$	74.122	22.57
1-Butanol	3.71	$C_4H_{10}O$	74.122	1.83
Acetoin	4.6	$C_4H_8O_2$	88.105	2.17
2,3-Butanediol, [R-(R*,R*)]-	6.85	$C_4H_{10}O_2$	90.121	1.41
2,3-Butanediol, [S-(R*,R*)]-	7.04	$C_4H_{10}O_2$	90.121	6.12
Oxime-, methoxy-phenyl-_	10.33	$C_8H_9NO_2$	151	3.70
2-Ethyl-1-hexanol	14.74	$C_8H_{18}O$	130.228	3.84
Undecane	15.74	$C_{11}H_{24}$	156.31	5.81
Decane, 2,4,6-trimethyl-	15.95	$C_{13}H_{28}$	184.361	1.24
Nonanal	17.57	$C_9H_{18}O$	142.24	2.99
6-Methyloctadecane	20.97	$C_{19}H_4O$	268.521	1.11
Decane, 2,3,5,8-tetramethyl-	23.56	$C_{14}H_3O$	198.388	1.16
Dodecane, 2,6,11-trimethyl-	23.76	$C_{15}H_{32}$	212.415	3.75
Tetradecane, 2,6,10-trimethyl-	32.36	$C_{17}H_{36}$	240.468	0.68
3-Trifluoroacetoxypentadecane	38.32	$C_{17}H_{31}F$	324.422	3.13
n-Hexadecanoic acid	40.77	$C_{16}H_{32}O_2$	256	1.64

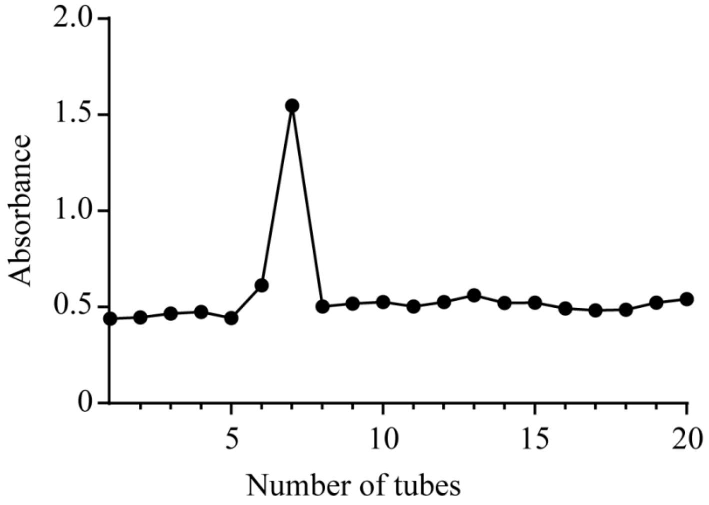

Supplementary Figure S1 Elution curve of crude protein on SepHadexG-100.
